# Elevated GM3 plasma concentration in idiopathic Parkinson’s disease: A lipidomic analysis

**DOI:** 10.1371/journal.pone.0172348

**Published:** 2017-02-17

**Authors:** Robin B. Chan, Adler J. Perotte, Bowen Zhou, Christopher Liong, Evan J. Shorr, Karen S. Marder, Un J. Kang, Cheryl H. Waters, Oren A. Levy, Yimeng Xu, Hong Bin Shim, Itsik Pe’er, Gilbert Di Paolo, Roy N. Alcalay

**Affiliations:** 1 Department of Pathology and Cell Biology, Columbia University Medical Center, New York, New York, United States of America; 2 Taub Institute for Research on Alzheimer’s Disease and the Aging Brain, Columbia University Medical Center, New York, New York, United States of America; 3 Department of Biomedical Informatics, Columbia University Medical Center, New York, New York, United States of America; 4 Department of Neurology, Columbia University Medical Center, New York, New York, United States of America; Karolinska Institutet, SWEDEN

## Abstract

Parkinson’s disease (PD) is a common neurodegenerative disease whose pathological hallmark is the accumulation of intracellular α-synuclein aggregates in Lewy bodies. Lipid metabolism dysregulation may play a significant role in PD pathogenesis; however, large plasma lipidomic studies in PD are lacking. In the current study, we analyzed the lipidomic profile of plasma obtained from 150 idiopathic PD patients and 100 controls, taken from the ‘Spot’ study at Columbia University Medical Center in New York. Our mass spectrometry based analytical panel consisted of 520 lipid species from 39 lipid subclasses including all major classes of glycerophospholipids, sphingolipids, glycerolipids and sterols. Each lipid species was analyzed using a logistic regression model. The plasma concentrations of two lipid subclasses, triglycerides and monosialodihexosylganglioside (GM3), were different between PD and control participants. GM3 ganglioside concentration had the most significant difference between PD and controls (1.531±0.037 pmol/μl versus 1.337±0.040 pmol/μl respectively; p-value = 5.96E-04; q-value = 0.048; when normalized to total lipid: p-value = 2.890E-05; q-value = 2.933E-03). Next, we used a collection of 20 GM3 and glucosylceramide (GlcCer) species concentrations normalized to total lipid to perform a ROC curve analysis, and found that these lipids compare favorably with biomarkers reported in previous studies (AUC = 0.742 for males, AUC = 0.644 for females). Our results suggest that higher plasma GM3 levels are associated with PD. GM3 lies in the same glycosphingolipid metabolic pathway as GlcCer, a substrate of the enzyme glucocerebrosidase, which has been associated with PD. These findings are consistent with previous reports implicating lower glucocerebrosidase activity with PD risk.

## Introduction

Parkinson’s disease (PD) is a common neurodegenerative disease, the burden of which is expected to grow as the current population ages. Numerous studies have described the contribution of aberrant lipid metabolism in a variety of neurodegenerative diseases, such as Alzheimer’s disease [1] and Huntington’s disease [2]. Similarly, lipid metabolism dysregulation may play a significant role in PD pathogenesis [3,4]. The pathological hallmark of PD is the accumulation of α-synuclein aggregates in Lewy bodies. The amphipathic α-helical domain in the N-terminus of α-synuclein mediates interaction with membrane phospholipids [3,5,6]. Biophysical evidence suggests that α-synuclein binds preferentially to acidic phospholipids over lipids with net neutral charge. Such interactions could potentially play a role in modulating the catalytic activity of various cytoplasmic lipid enzymes as well as lysosomal lipases that are dependent on negatively charged lipids [7,8]. Further support of the importance of lipid dysregulation in PD is driven by genetic studies, where mutations in glucocerebrosidase (*GBA*) are strongly associated with PD [9]. *GBA* encodes the lysosomal enzyme β-glucosidase (GCase), which hydrolyzes glucosylceramide and glucosylsphingosine to ceramide and sphingosine, respectively, and plays a key role in the glycosphingolipid metabolic pathway. Diminished GCase activity as a result of homozygous or compound heterozygous mutations in *GBA* leads to Gaucher disease, the most common genetic lysosomal storage disease. In addition to *GBA*, other lysosomal proteins have been associated with PD, e.g., acid sphingomyelinase [10] (*SMPD1*, deficient in Niemann-Pick disease types A and B) and LIMP-2 [11], a GCase transporter. Furthermore, several classical genetic risk factors of PD are associated with normal lipid function and metabolism as well as membrane trafficking, particularly with the endolysosomal pathway [12,13]. For example, *LRRK2* has been linked to endolysosomal/autophagic function [14], while *Parkin* and *PINK1* have been associated with mitochondria, the major cellular stations controlling lipid synthesis and metabolism [15]. Mutations in the retromer protein VPS35, which is part of a complex involved in the delivery of lysosomal enzymes to lysosomes via retrograde transport of the mannose 6-phosphate receptor, has also been linked to PD neurodegeneration [16]. Further, a recent study showed an association between apolipoprotein A1 (ApoA1) and several clinical variables in PD, including earlier age-at-onset [17].

Because of the growing evidence of lipid involvement in PD pathogenesis, we hypothesized that plasma lipids may be altered in PD. A blood-based biomarker would be attractive because it could be accessed easily and inexpensively in a clinical setting. Several published studies have analyzed plasma lipids and apolipoprotein as a way to determine the severity and duration of PD progression [17–20]. In this study, we applied unbiased lipidomic analysis to plasma collected from idiopathic PD patients and controls to test whether specific lipid species differed between the two groups.

## Materials and methods

### Participants and clinical evaluation

The study included 150 PD participants and 100 controls who participated in the ‘Spot’ study [21,22]. In brief, the Spot study included PD patients and genetically unrelated controls (mostly spouses) from the Center for Parkinson’s Disease at Columbia University Medical Center in New York, NY, recruited between 2010–2016. In the current study we included 150 (75 men and 75 women) consecutive PD Spot participants and 100 controls (50 men and 50 women), frequency-matched by gender, age and statin use to the PD cases. All participants have been fully sequenced for *GBA* mutations and screened for the *LRRK2* G2019S mutation. Only non-carriers (except for one *GBA*/PD case, whose exclusion did not change the results) were included. Subjects were non-fasting. Information on demographics, medical history, medication, family history of PD [23], the Unified Parkinson’s Disease Rating Scale (UPDRS) in the “on” state and the Montreal Cognitive Assessment (MoCA)[24] were collected from all participants. A 10cc EDTA tube was used to collect blood which was centrifuged and aliquoted to 1cc plasma aliquots within 60 minutes of collection. Samples were stored in a -80°C freezer until processing. All study procedures were approved by the Columbia University IRB, and all participants signed informed consent.

### Analysis of lipids using high performance Liquid Chromatography-Mass Spectrometry (LC-MS)

Plasma lipid extracts were prepared by modifying previously published lipid extraction protocols [25]. Briefly, each lipid extract sample was prepared from 100μl of plasma that was mixed with 900μl of chloroform-methanol (5:4 v/v), followed by 200μl of 1M KCl. The mixture was spiked with appropriate internal standards, vigorously vortexed and centrifuged for phase separation, after which the organic lower phase was collected and dried using a speed vacuum. The lipid extracts were analyzed using a 6490 Triple Quadrupole LC-MS system (Agilent Technologies, Santa Clara, CA). Glycerophospholipids and sphingolipids were separated with normal-phase HPLC as described before [25], with a few modifications. An Agilent Zorbax Rx-Sil column (inner diameter 2.1 x 100 mm) was used under the following conditions: mobile phase A (chloroform:methanol:1 M ammonium hydroxide, 89.9:10:0.1, v/v) and mobile phase B (chloroform:methanol:water:ammonium hydroxide, 55:39.9:5:0.1, v/v); 95% A for 2 min, linear gradient to 30% A over 18 min and held for 3 min, and linear gradient to 95% A over 2 min and held for 6 min. Sterols and glycerolipids were separated with reverse-phase HPLC using an isocratic mobile phase as before [25] except with an Agilent Zorbax Eclipse XDB-C18 column (4.6 x 100 mm). The LC separation of lipid extracts prior to MS analysis is an important component of the analysis as it prevents signal suppression of low abundance lipid species such as phosphatidic acid (PA), bis(monoacylglycero)phosphate (BMP) and GM3 by lipids that are typically found in higher abundance, such as phosphatidylcholine (PC) and phosphatidylethanolamine (PE). In total, we measured 520 unique lipid species, covering a broad spectrum of 39 lipid subclasses. The lipid species are listed in S1 Table.

Quantification of endogenous lipid species was accomplished using multiple reaction monitoring (MRM) transitions that were developed in earlier studies [25,26] in conjunction with referencing to the signal intensities of known quantities of internal standards: PA 14:0/14:0, PC 14:0/14:0, PE 14:0/14:0, phosphatidylglycerol (PG) 15:0/15:0, phosphatidylinositol (PI) 12:0/13:0, phosphatidylserine (PS) 14:0/14:0, BMP 14:0/14:0, acyl phosphatidylglycerol (APG) 14:0/14:0/14:0, lysophosphatidylcholine (LPC) 13:0, lysophosphatidylethanolamine (LPE) 14:0, lysophosphatidylinositol (LPI) 13:0, ceramide (Cer) d18:1/17:0, sphingomyelin (SM) d18:1/12:0, dihydrosphingomyelin dhSM d18:0/12:0, galactosylceramide (GalCer) d18:1/12:0, sulfatide (Sulf) d18:1/12:0, glucosylceramide (GlcCer) d18:1/12:0, lactosylceramide (LacCer) d18:1/12:0, D7-cholesterol, cholesterol ester (CE) 17:0, monoacylglycerol (MG) 17:0, 4methyl 16:0 diether diacylglycerol (DG), D5-triacylglycerol (TG) 16:0/18:0/16:0 and Lipidomix HP(25) (Avanti Polar Lipids, Alabaster, AL). We were not able to procure lipid standards for some lipid species that we measured, including GM3 and globotriaosylceramide (Gb3). In such cases, we referenced these lipid species to the internal standards that were closest to it in the elution gradient. The lipid standards we used to match specific lipid species we measured are listed in S1 Table. The detection of hydroxylated cholesterol metabolites, such as 24(S)- or 27-hydroxycholesterol, is not reliable with our current LCMS methodology, and those lipids were not included.

The concentrations of individual lipid species are presented as pmol/μl of plasma sample. As an alternate quantification, we also calculated lipid concentrations as mol% of the total lipid measured to correct for the differences in total lipid amount between samples. Mol% values were calculated by dividing individual lipid concentration by the total lipid concentration (excluding cholesterol and cholesterol ester) of each sample. Both values are presented in S2 and S3 Tables. The average concentration of individual lipid species presented were obtained by averaging across all samples with the same PD status and/or gender and calculating the standard error of mean. The lipidomic team was blinded to clinical data, including PD status, when analyzing the samples.

#### Annotation of lipid species

Glycerophospholipids, lyso phospholipids and diacylglycerol (DAG) were annotated as <lipid abbreviation> <total fatty acyl chain length>:<total number of unsaturated bonds>. Sphingolipids were annotated as <lipid abbreviation> <sphingoid base residue>/<fatty acyl residue>. Triacylglycerol (TAG) was annotated as <TAG> <total fatty acyl chain length>:<total number of unsaturated bonds>(<diacylglycerol residue>/<fatty acyl residue>). CE was annotated as <CE>-<fatty acyl residue>.

### Statistical analysis

Demographics and clinical characteristics were compared between PD and control participants by Student's t test, Chi Square and the Fisher Exact test as appropriate. The lipid measures in both pmol/μl and mol% were quantile normalized, and each lipid was in-turn fitted with a logistic regression model (R version 3.2.2, package ‘preprocessCore’) with PD status as the dependent variable, lipid measure as independent variables while controlling for age, sex, body mass index (BMI), and statin usage. For each lipid, we evaluated the statistical significance of residuals for predicting PD status based on Wald’s χ^2^-test (raw p-value). We then evaluated q-values, controlling for the false discovery rate at a level of 0.05 (across all the measurements of either lipid subclass totals or the specific lipid species, R package ‘qvalue’) [27]. It should be noted that we conducted a preliminary study with small sample size including samples from different Spot participants, where we identified significant differences in GM3 concentration between PD and controls (data not shown); however, we present here conservative statistical analysis, not taking into account our primary hypothesis of differential GM3 concentration in PD and controls.

In the classification analysis, the samples were randomly separated in a 90/10 split into development and validation cohorts. Quantile normalization was applied separately to the lipid measures of both cohorts and a support vector machine with a radial basis kernel function (R package ‘e1071’) was trained with the development cohort and evaluated with the validation cohort. Features included 20 selected lipid species that lie in the GCase pathway, including GM3 and GlcCer, as well as the controlled variables listed above. We used a random resampling procedure to estimate the expected area under the receiver operating characteristic (ROC) curve.

## Results

### Characteristics of participants

Demographics and clinical characteristics of PD and control participants are compared in Table 1. By design, there was no significant difference in gender, age or statin use between PD and control participants. PD and control participants also had similar median BMI (PD = 25, controls = 25) and median MoCA scores (PD = 26, controls = 27). The median disease duration of PD participants was 4 years (range 0–27 years), median “on” UPDRS-III (motor) score was 15 (range 0–48), and median levodopa equivalent dose was 400mg (range 100-1500mg).

**Table 1:** Demographic and Parkinson’s disease characteristics of the cohort in this study.

	All participants^1^		Male^1^		Female^1^	
	PD (n = 150)	Control (n = 100)	PD (n = 75)	Control (n = 50)	PD (n = 75)	Control (n = 50)
Characteristics	Mean (Min, Max)	Mean (Min, Max)	Mean (Min, Max)	Mean (Min, Max)	Mean (Min, Max)	Mean (Min, Max)
Age	66.5 (45, 87)	66.1 (44, 90)	66.0 (45, 86)	67.7 (47, 90)	67.0 (45, 87)	64.6 (44, 83)
BMI	25.7 (17, 50)	25.7 (16, 40)	26.6 (19, 50)	26.9 (19, 40)	24.8 (17, 39)	24.5 (16, 37)
Number of participants using statins (%)	31 (20.6%)	22 (22.0%)	12 (16.0%)	13 (26.0%)	19 (25.3%)	9 (18.0%)
Duration of PD in years	5.7 (0, 27)		5.5 (0, 22)		5.9 (0, 27)	
Levodopa Equivalent Dose in mg	462.7 (0, 1500)		511.3 (0, 1500)		414.2 (0, 1100)	
UPDRS I+II	9.4 (0, 30)	0.8 (0, 9)	10.9 (0, 30)	0.9 (0, 9)	7.9 (0, 24)	0.8 (0, 4)
UPDRS III	16.3 (0, 48)	1.7 (0, 12)	18.2 (3, 48)	1.7 (0, 12)	14.4 (0, 40)	1.7 (0, 10)
MoCA	25.7 (12, 30)	27.0 (20, 30)	25.3 (12, 30)	26.7 (20, 30)	26.1 (18, 30)	27.4 (23, 30)

1. Age, BMI, number of participants using statins at the time of recruitment and MoCA score were similar between PD and controls both in the analyses including the entire cohort and in analyses stratified by gender.

BMI: body mass index; UPDRS: Unified Parkinson’s Disease Rating Scale; MoCA: Montreal Cognitive Assessment.

### Identification of lipid biomarkers in PD patient plasma

The extracted plasma lipid samples were analyzed blinded to clinical characteristics, using the LC-MS methodology described above. Of the 39 subclasses of lipids tested, including 520 specific lipid species, we identified two lipid subclasses, triglycerides and GM3, and 33 lipid species that were significantly different between PD patients and healthy controls. The list of all significant lipid species is included in S2 Table. Of note, in addition to GM3 and TG changes, 5 Cer species (i.e., GCase product) were significantly lower in PD than controls.

**Fig 1:**
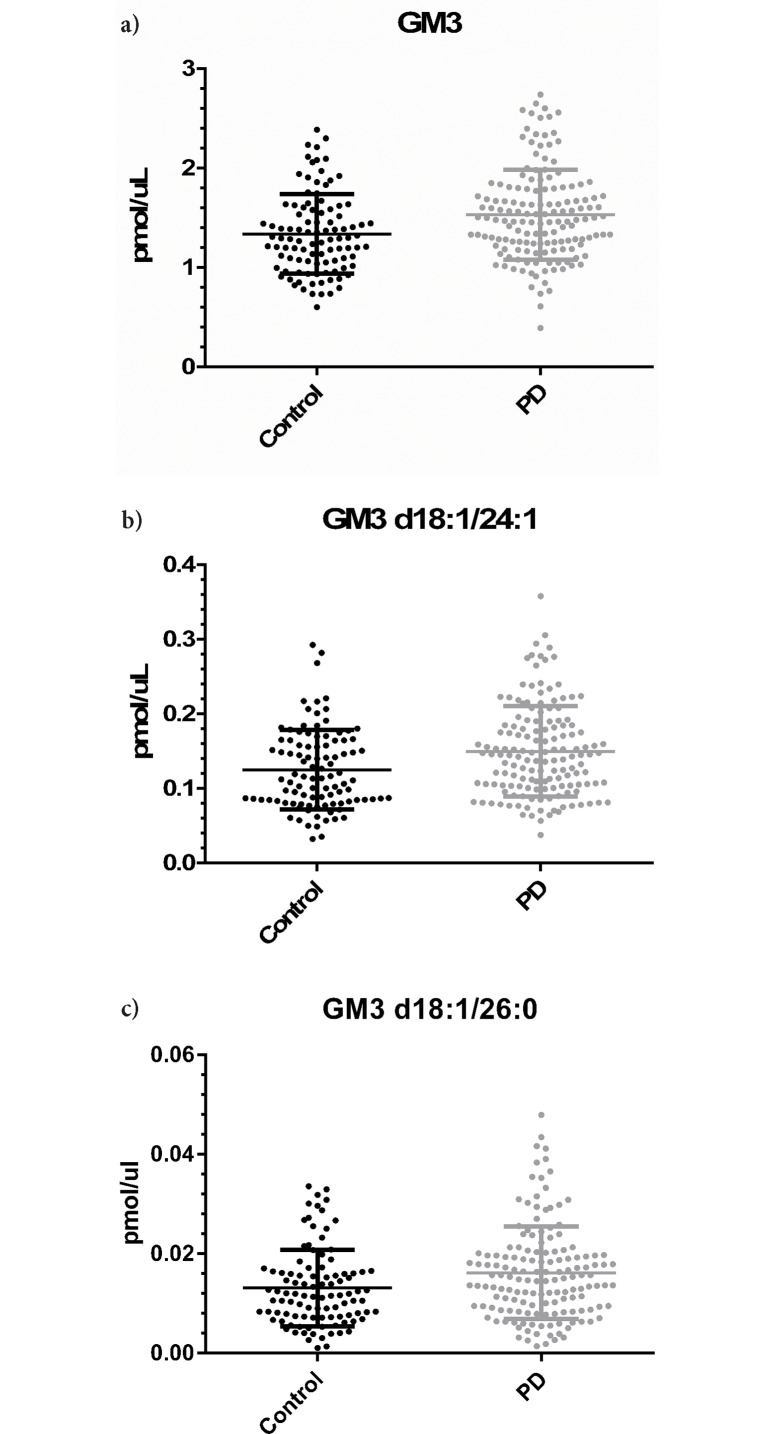
Higher levels of GM3 in PD patients compared to controls. Scatter plots are shown for total GM3 (A), and for the GM3 species GM3 d18:1/24:1 (B) and GM3 d18:1/26:0 (C).

The most striking differences between controls and PD were in GM3 (1.337±0.040 pmol/μl vs. 1.531±0.037 pmol/μl for controls and PD respectively; p-value = 5.96E-04; q-value = 0.048; when normalized to total lipid: p-value = 2.890E-05; q-value = 2.933E-03; Fig 1A). The difference in GM3 concentration between controls and PD was the result of higher levels of multiple GM3 species, of which the most prominent were GM3 d18:1/24:1 (0.125±0.005 pmol/μl vs. 0.149±0.005 pmol/μl; p-value = 0.0012; q-value = 0.048; when normalized to total lipid: p-value = 4.180E-05; q-value = 2.933E-03; Fig 1B) and GM3 d18:1/26:0 (0.013±0.001 pmol/μl vs. 0.016±0.001 pmol/μl; p-value = 3.307E-04; q-value = 1.392E-02; when normalized to total lipid: p-value = 1.571E-02; q-value = 4.886E-02; Fig 1C). We also found that TG levels were about 25% lower in PD patients compared to controls (p-value = 0.0041; q-value = 0.0497; when normalized to total lipid: p-value 5.837E-03; q-value = 2.382E-02; S2 Table); however, because of a greater variance (likely reflecting the fact that participants were not fasting for this study), statistical significance of the differences in TG plasma levels was not as strong as differences in GM3 levels.

### Effects of sex on plasma lipid levels

A previous study of more than 1000 participants demonstrated different plasma levels of several sphingolipid subclasses between males and females [28]. We therefore stratified our analyses by sex. The GM3 concentration differences between PD and controls were stronger in males than in females (Males: 1.288±0.052 pmol/μl versus 1.539±0.047 pmol/μl for controls and PD respectively; p-value = 0.0038; q-value = 0.038; when normalized to total lipid: p-value = 1.472E-04; q-value = 1.412E-02. Females: 1.386±0.060 pmol/μl versus 1.523±0.057 pmol/μl for controls and PD respectively; p-value = 0.03; q-value > 0.05; when normalized to total lipid: p-value = 6.051E-04; q-value > 0.05). In addition to GM3, male PD participants showed other significant lipid subclass alterations, including higher concentrations of N-Acyl plasmalogen phosphatidylethanolamine (NAPEp, p-value = 0.00827; q-value = 0.04848; when normalized to total lipid: p-value = 1.55E-03; q-value = 1.740E-02), and lower amounts of DG (p-value = 0.0030; q-value = 0.0349; when normalized to total lipid: p-value = 1.251E-02; q-value = 4.475E-02) and TG (p-value = 0.0014; q-value = 0.0326; when normalized to total lipid: p-value = 2.239E-03; q-value = 2.113E-02). In contrast, female PD patients showed no significant differences in any lipid subclass compared to female controls.

**Fig 2:**
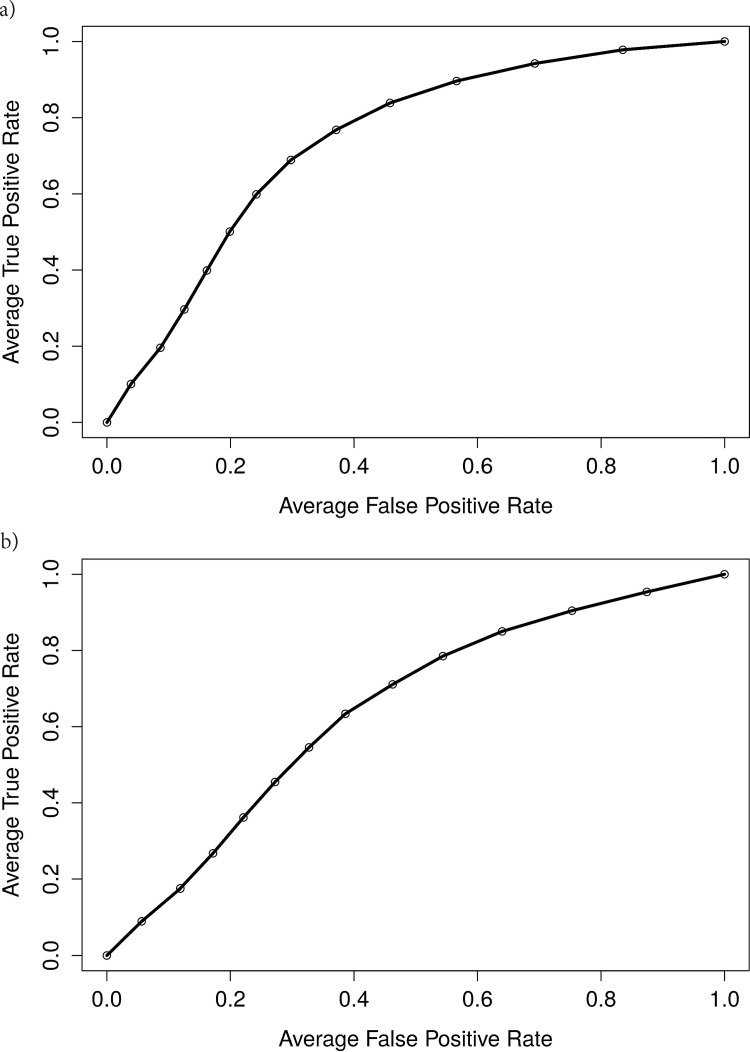
GM3 levels are more predictive of male PD status. The receiver operating characteristic (ROC) curves for male (A) and female (B) participants in our studies are shown. Their respective AUC is shown with the graph.

A receiver operating curve (ROC) analysis using non-linear classifier was conducted to evaluate the utility of 20 selected lipid species (normalized to total lipid), consisting of GM3 and GlcCer (Table 2), in discriminating patients with PD from healthy controls. These two lipid subclasses were selected based on the knowledge that GCase activity is a risk factor for the development of PD [29,30]. The area under the curve (AUC) was found to be 0.742 for men (Fig 2A) and 0.644 for women (Fig 2B).

**Table 2:** GM3 and GlcCer lipid species that were included in the ROC analysis.

Lipid	Control, mol% (pmol/μl)	PD, mol% (pmol/μl)	p-value^1^	q-value^1^
GlcCer d18:1/16:0	0.066±0.002 (0.467±0.017)	0.076±0.002 (0.514±0.014)	0.002577	0.018696
GlcCer d18:1/16:1	0.00033±0.00001 (0.0023±0.0001)	0.00038±0.00001 (0.0025±0.0001)	0.003954	0.019907
GlcCer d18:1/18:0	0.0084±0.0003 (0.059±0.002)	0.0096±0.0003 (0.065±0.002)	0.008245	0.030438
GlcCer d18:1/18:1	0.00088±0.00003 (0.0062±0.0002)	0.00104±0.00003 (0.0070±0.0002)	0.002438	0.018658
GlcCer d18:1/20:0	0.0088±0.0003 (0.062±0.002)	0.0103±0.0003 (0.069±0.002)	0.001494	0.016639
GlcCer d18:1/20:1	0.00039±0.00002 (0.0027±0.0001)	0.00044±0.00001 (0.0030±0.0001)	0.017936	0.040584
GlcCer d18:1/22:0	0.087±0.003 (0.608±0.023)	0.098±0.003 (0.659±0.019)	0.011068	0.035833
GlcCer d18:1/22:1	0.0046±0.0002 (0.032±0.001)	0.0050±0.0002 (0.034±0.001)	0.017187	0.039446
GlcCer d18:1/24:0	0.116±0.004 (0.809±0.031)	0.134±0.004 (0.896±0.027)	0.004331	0.020517
GlcCer d18:1/24:1	0.082±0.003 (0.566±0.022)	0.097±0.003 (0.651±0.022)	0.001302	0.016639
GM3 d18:1/16:0	0.039±0.002 (0.262±0.015)	0.047±0.002 (0.308±0.014)	0.001581	0.016639
GM3 d18:1/16:1	0.0025±0.0002 (0.017±0.001)	0.0030±0.0001 (0.020±0.001)	0.00201	0.01762
GM3 d18:1/18:0	0.031±0.001 (0.214±0.006)	0.035±0.001 (0.236±0.006)	0.000605	0.015612
GM3 d18:1/18:1	0.0045±0.0002 (0.031±0.001)	0.0054±0.0002 (0.036±0.001)	0.001579	0.016639
GM3 d18:1/20:0	0.020±0.001 (0.143±0.006)	0.023±0.001 (0.157±0.005)	0.012061	0.037342
GM3 d18:1/22:0	0.036±0.001 (0.255±0.011)	0.042±0.001 (0.287±0.010)	0.001451	0.016639
GM3 d18:1/22:1	0.0087±0.0004 (0.061±0.003)	0.0105±0.0003 (0.071±0.003)	0.000331	0.013916
GM3 d18:1/24:0	0.027±0.001 (0.190±0.008)	0.033±0.001 (0.222±0.008)	0.000873	0.015612
GM3 d18:1/24:1	0.018±0.001 (0.125±0.005)	0.022±0.001 (0.149±0.005)	0.000042	0.002933
GM3 d18:1/26:0	0.0019±0.0001 (0.013±0.001)	0.0024±0.0001 (0.016±0.001)	0.001154	0.016639

1. The p- and q-values shown here were calculated using lipid concentrations in mol%.

## Discussion

In this study, we report a comparison of 520 plasma lipid species from 39 different lipid subclasses in 150 PD cases and 100 controls, non-carriers of *GBA* or *LRRK2* G2019S mutations. Our key finding is the significantly higher GM3 plasma concentration in PD cases compared to controls.

GM3 lipids are considered the simplest members of the ganglioside family, containing three monosaccharide groups attached to a ceramide backbone. They serve as precursors to complex gangliosides of the a- and b-series that are abundant in the brain [31]. GM3 and other gangliosides are synthesized in the Golgi apparatus before their delivery to the plasma membrane, where they play various important roles in cellular signaling. They are then internalized and targeted via the endocytic pathway to the lysosome, where they are metabolized into ceramide and monosaccharide through a pathway that involves GCase. Reduced GCase activity in the central nervous system [32–34] and in the periphery [35] has been associated with PD in many but not all [36,37] studies. The mechanism of the association between reduced GCase activity and PD is unknown, but one proposed mechanism would be alteration of lipids metabolized by GCase. Indeed, our finding of elevated GM3 levels in PD compared to controls supports this hypothesis. Further support of a potential link between GCase activity, elevated GM3 and PD may be stipulated from Gaucher patients, who not only have a significantly greater risk of developing PD [30], but also show elevated plasma GM3 concentrations [38]^,^[39].

Our data partially corroborates the work of Mielke et al. [19], where other lipids in the GCase pathway, Cer and monohexylceramide (includes both GlcCer and GalCer), were found at a higher level in PD patients than controls. An important difference between our work and theirs pertains to the scope of analytical coverage. Mielke and colleagues focused on limited sphingolipid analysis that did not include GM3 and had a smaller sample size (n = 57). Despite these differences, both studies identified lipid metabolites that are closely linked to the GCase pathway (Fig 1B and Table 2), thereby further strengthening the association of the GCase pathway and PD pathogenesis.

While we cannot determine the relationship between GM3 levels in plasma and the brain, our findings of elevated GM3 in PD are consistent with the neuropathological literature. A study of 118 autopsies of subjects age 20 to 100, demonstrated increasing proportions of GM3 with age[40], and two neuropathological studies showed a trend of higher GM3 in basal ganglia of PD brains compared to controls[41,42].

The potential mechanism by which altered GM3 levels may be associated with PD was investigated in a few models. Di Pasquale and colleagues identified a ganglioside-binding domain (GBD) in α-synuclein, which has marked preference to GM3. This GBD includes residue E46, which is mutated in a familial form of PD (E46K). The deleterious effect of the mutation (in forming channels) was reversed in vesicles enriched with GM3[43]. Conversely, Grey and colleagues isolated exosomes from neuroblastoma cells, and using mass spectrometry identified different phospholipid classes in the exosomes. When they prepared vesicles from corresponding pure lipids, they observed that GM3 (as well as GM1) accelerated α-synuclein aggregation, as opposed to the rest of phospholipids, which slowed down aggregation [44]. However, observations from these two papers are likely to be more relevant for the aggregation of extracellular pools of α-synuclein than for aggregation of intracellular α-synuclein. This is important because aggregation of extracellular α-synuclein may be involved in the spreading of pathology during disease progression in PD [45].

Our study has some limitations. First, the degree of natural variation in blood lipids between and within individuals and the reference population is not well understood or documented. Plasma lipid composition variability is driven by a complex interplay of factors including genetics, diet, environment, and even autonomous circadian clocks [28,46,47], and such fluctuations complicate comparative studies. Our samples were collected from non-fasting participants. Dietary lipids have been shown to be incorporated into liver lipids and subsequently lipoprotein particles, which contribute to plasma lipid composition [48]. However, a recently published study showed that only certain species of phosphatidylethanolamine (PE) and phosphatidylcholine (PC), but not GM3, are affected by food intake and time of sample collection [47]. Replicating this study in samples collected from fasting cohorts is needed to further validate our GM3 observation. Second, establishing reference levels of specific lipids is essential before such lipids can be used in a clinical setting. In our study, we used surrogate standards to reference GM3 levels because no suitable synthetic GM3 standards exist. Given the evidence provided here for GM3 as a PD biomarker candidate, GM3 standards for future studies are required. However, the difference we found in GM3 concentration between PD and control is 14.5% (Fig 1). Thus GM3 levels cannot be used alone for diagnostic purposes and need to be combined with other measurements to have diagnostic value. The major strengths of our study include the largest lipidomic analysis reported to date in PD, and the carefully phenotyped cohort. We further excluded potential confounders by including only PD cases and controls without *GBA* or *LRRK2* G2019S mutations.

In summary, our findings highlight the potential role of GM3 in idiopathic PD. Future studies should not only examine the lipid panel used in this study, but also include other complex gangliosides like GM2 and GD3, which are derived from GM3 in its biosynthetic pathway. Exploring interactions between these glycosphingolipids and α-synuclein as well as replicating our findings in neuropathological samples is necessary to better understand the pathophysiology of GM3 in idiopathic PD.

## Supporting information

S1 Table The lipid species measured in this analysis (and their abbreviations) along with the lipid standards used to match the specific lipids. (XLSX)

S2 Table The concentration of lipid species significantly different between PD and controls (expressed both in pmol/μl and in mol%). (XLSX)

S3 Table The concentration of individual lipid species in each participant (expressed both in pmol/μl and in mol%). (XLSX)
